# Against tiebreaking arguments in priority setting

**DOI:** 10.1136/jme-2023-108972

**Published:** 2023-06-08

**Authors:** Borgar Jølstad, Erik Gustavsson

**Affiliations:** 1 The Health Services Research Unit – HØKH, Akershus Universitetssykehus HF, Lorenskog, Norway; 2 Centre for Medical Ethics, Institute of Health and Society, University of Oslo, Oslo, Norway; 3 Centre for Applied Ethics, Department of Culture and Society, Linkopings Universitet, Linkoping, Sweden; 4 National Centre for Priorities in Health, Department of Health, Medicine and Caring Sciences, Linkopings Universitet, Linkoping, Sweden

**Keywords:** Decision Making, Ethics

## Abstract

Fair priority setting is based on morally sound criteria. Still, there will be cases when these criteria, our primary considerations, are tied and therefore do not help us in choosing one allocation over another. It is sometimes suggested that such cases can be handled by tiebreakers. In this paper, we discuss two versions of tiebreakers suggested in the literature. One version is to preserve fairness or impartiality by holding a lottery. The other version is to allow secondary considerations, considerations that are not part of our primary priority setting criteria, to be decisive. We argue that the argument for preserving impartiality by holding a lottery is sound, while the argument for using tiebreakers as secondary considerations is not. Finally, we argue that the instances where a tiebreaker seems necessary are precisely the situations where we have strong reasons for preferring a lottery. We conclude that factors that we consider valuable should all be included among the primary considerations, while ties should be settled by lotteries.

## Introduction

When allocating healthcare resources, we strive to base our choices on morally relevant factors. There are arguments about the relevance of cost, benefit, age, personal responsibility, adaptation, illness severity, basic needs and a host of other candidates that arguably have moral relevance for priority setting. Although a healthcare system may have principles for priority setting in place there will be cases when these principles cannot provide an answer to whether one allocation should be chosen rather than another. When it comes to such cases, it is often suggested that certain concerns could play the role of tiebreakers. That is, a tiebreaker is a factor that is decisive when there are no other relevant differences between groups or individual patients. Tiebreakers can, for example, be found in numerous of the guidelines for priority setting developed during the COVID-19 pandemic.^1–4^ Among the 26 US state guidelines surveyed by Piscitello *et al* 3 about half of them include tiebreakers. Age, status as a healthcare worker, first come, first served and lotteries, have all been suggested as tiebreakers. However, tiebreakers are also suggested outside pandemic guidelines. For example, Thornton^5^ has argued that individual responsibility could work as a tiebreaker and decide which patient gets a liver transplant when these patients are equal in terms of medical need and waiting time. In this paper, we distinguish between two ways in which tiebreakers can be characterised and argue that while one of them is uncomplicated the other is problematic both in theory and in practice.

The paper proceeds as follows. First, we will discuss what is meant by there being a tie and what this tells us about the importance of tiebreakers. Second, we will describe two types of tiebreaking arguments: those concerned with preserving fairness or impartiality and those concerned with tiebreakers as secondary (rather than primary) considerations. Third, we discuss using tiebreakers as a secondary consideration with a special focus on the challenge of explaining why a factor would be morally relevant only in the instance of a tie. Finally, we argue that the instances where a tiebreaker seems necessary are precisely the situations where we have strong reasons for preferring a lottery.

## When do we have a tie?

Let us introduce some terminology here. We will follow Altman^6^ and use the term primary considerations to refer to the values that have been deemed morally relevant in the first place for ranking options for priority setting in a healthcare system. In the argument made by Thornton, this would be medical need and waiting time. Tiebreakers are considerations that are not included among the primary considerations but are allowed to break ties. They are thus secondary considerations, as exemplified by moral responsibility in Thornton’s framework. A tie occurs when there are no morally relevant differences between options in terms of primary considerations. That cases should be treated alike when there are no relevant moral differences is one of the least controversial tenets of ethics.^1^ What ‘morally relevant’ means will depend on our theoretical commitments. If, for example, we are committed to prioritise based on cost-effectiveness in terms of time spent in better health, morally relevant differences can plausibly be as small as brief time differences or very small differences in health. The other end of the spectrum may be illustrated by the Australian priority guidelines for clinical care in pandemics described by Dawson *et al*.^4^ The guidelines define a ‘high priority’ and ‘low priority’ group based on likelihood of recovery and benefiting from, for example, ICU treatment. They then suggest that previous disadvantage and status as a healthcare professional can work to break ties within the ‘high priority’ group. In this framework, morally irrelevant differences can be ‘larger’; anyone considered to be in the ‘high priority’ group is on the same footing, that is, they are tied. Hence, how common a tie is, and how practically important a tiebreaker is, depends on our theoretical commitments to moral relevance. With guidelines based on some fine-grained measure of good life-years ties will, assuming we have perfect information, rarely occur because there will always be some small difference between options. However, if decision-makers use the Australian guidelines, they are bound to happen more often. Tiebreakers will, all else being equal, play larger roles and de facto be more important in a system where ties are more frequent.

## Two kinds of tiebreakers

There are at least two prima facie reasons for admitting tiebreakers in our priority system scheme. First off, we sometimes need a tiebreaker: there are cases where there seems to be no reason whatsoever to make one choice rather than some other. This can either be due to situational factors, such as not having access to the information that would allow us to make a choice without a tiebreaker, or due to there being an actual tie. In these situations, securing fairness or impartiality is an important motivating factor. Second, we may think that a feature has some importance, but not enough to qualify as a primary consideration. For instance, we might be unsure of the status of personal responsibility as a priority setting criterion, but we may want to give it some weight in our priority setting system. Allowing personal responsibility to play the role of a tiebreaker may be a way of making a modest concession to the moral relevance of responsibility.

### Securing fairness or impartiality by holding a lottery

In situations where people have an equal claim, such as in ties, holding a lottery is an attractive option.^2^ The value of lotteries can be grounded in reasons such as impartiality, respect for the separateness of persons, fairness and the moral value of chances (as opposed to realised options).^3^ Broome,^7^ for instance, argues that lotteries mitigate unfairness by giving everyone a ‘surrogate satisfaction’: a chance at the good to be distributed. Importantly, one can argue for the attractiveness of a lottery even from a strictly utilitarian perspective. Tännsjö^8^ has, for example, argued that a lottery can be used to break a tie when people have the same claim on a resource, in order to ensure impartiality. For example, we may want to ensure that we do not choose between two individuals on discriminatory grounds.

Lotteries may be seen as expressions of fundamental impartiality or equality. In cases where there are no morally relevant differences between two options they are, by definition, morally equal. The lottery then gives both individuals the same probability, which is an expression of fairness or impartiality. However, there is another sense of tiebreakers that account for them in a seemingly different way, namely in terms of primary and secondary considerations.

### Primary and secondary considerations

Recall Thornton^5^ who discusses the importance of timely decision-making when two individuals need a liver transplant but there is only one liver available for donation. She argues that the primary considerations should be medical need or waiting time. However, sometimes these factors are tied. Thornton argues that when there is a tie between two individuals in this sense, individual responsibility should be used as a tiebreaker. The point of ascribing this role to responsibility is not to secure impartiality, but rather to give individual responsibility its due as a relevant factor for priority setting decisions. Individual responsibility is thus a secondary consideration in Thornton’s framework.

Since a tiebreaker is only relevant when there is a tie, it follows that any relevant difference among primary considerations trumps the tiebreaker. It is thus clear that a tiebreaker as a secondary consideration either has a value that is infinitesimal, or that its value is lexically subordinated to the primary considerations. Either of these options allow us to make sense of tiebreakers as a secondary consideration. For the purposes of our argument, we will concern ourselves with the lexical interpretation.^4^ To say that a primary value X takes lexical priority over a tiebreaker value Y is to say that any difference between individuals (or groups) with regard to X takes priority over any difference between these individuals (or groups) with regard to Y.

Tiebreakers as secondary considerations have been employed in real world priority setting during the COVID-19 pandemic. One example comes from the aforementioned Australian pandemic priority setting guidelines^4^: when deciding on whom from the ‘high priority group’ to prioritise, the authors suggest considering previous disadvantage and being a medical professional as potential tiebreakers. The considerations about whether one qualifies into the ‘high priority group’ are primary considerations, but previous disadvantage or status as a healthcare worker are considered important enough to qualify as a secondary consideration. In the USA, at least three standards for priority setting during ventilator-shortages operate with age as a tiebreaker.^6^ Interestingly, they all argue for the inclusion of age as a tiebreaker on the grounds that age is morally important; younger people have not yet had the opportunity to go through the various stages of life. Here the status of age as a secondary consideration is particularly salient; while age is considered morally important, it is relegated to play the role of a tiebreaker due to, perhaps, being more contentious than the primary considerations.

Tiebreakers as secondary considerations give rise to a puzzle. Why does the tiebreaker, if it represents a genuine moral concern, only matter in ties? In the next section, we turn to various motivations for tiebreakers as secondary considerations in priority setting.

## Motivating tiebreakers as secondary considerations

To provide a justification for tiebreakers as secondary considerations, only suitable for breaking ties, is challenging. On the one hand, a tiebreaker value is a genuine value providing reasons for acting, but on the other hand, it is not important enough to be included among the primary considerations.

Note that this puzzle about tiebreakers is different from the claim that some factor may be morally relevant in relation to some agents but not others. For example, holding patients responsible for some self-inflicted conditions may be considered less problematic when patients have sufficient control over their behaviour as compared with patients who do not. Here, we would have a factor that is relevant in some situations but not others, and that in this respect is like tiebreakers.^5^ However, there is an important difference: allowing a factor to be decisive in this manner is clearly motivated by morally relevant differences. We can, for example, argue that applying considerations of responsibility to children is too harsh as they may not have the relevant kind of control over their behaviour. Tiebreakers should also be morally motivated.

One way in which such a position could be motivated would be to say something along the following lines. Let us employ age as an example. We may believe that there are several reasons to take age into account in priority setting decisions. For example, the US guidelines discussed by Altman^6^ consider the opportunity to experience life stages such a reason. However, we may also believe that there are good reasons to not take age into account in these decisions, for example, the threat of ageism as discussed in the same guidelines. Furthermore, we may believe that the reasons that count in favour of taking age into account carry slightly more weight than the reasons that count against doing so. Therefore, we may conclude that age should be given some minimal weight in priority setting decisions and the way to do that is to use age as a tiebreaker. The problem is that this account lacks an argument for why the tiebreaking factor should be included as a secondary consideration, rather than among the primary considerations but with comparatively less weight ascribed to it. A lexical priority ordering should be clearly motivated in some manner to be plausible. Rawls, for instance, arrived at the difference principle by way of an argument from the veil of ignorance; with minimal information, the leximin decision procedure is the most rational.^9^ The lexical ordering is thus clearly motivated. The arguments presented in the mentioned guidelines^6^ and by Thornton^5^ motivate the idea that the relevant tiebreakers represent value that, due to being contentious, should be given some but not too much weight in priority setting. But why not simply include it among the primary considerations but ascribe less weight to it? What reasons do we have for claiming that the tiebreaker, an independent value, only makes a difference in ties? There should seemingly be some limit to how unimportant something can be, while still being valuable for its own sake. We call this the first tiebreaker problem.

*First tiebreaker problem*: if tiebreakers matter when there is a tie, why do they not matter in other situations?

This problem seems especially poignant given that lexicality is not the only way of making a modest concession to the importance of the tiebreaking factor. It would be, at least theoretically, unproblematic to include the factor among the primary considerations in a discounted manner.^6^ This would result in the tiebreaker value behaving like other values, while still rarely being decisive.

One could object that while we have theoretical reasons for rejecting tiebreakers, we may have pragmatic reasons for adopting them. Since our estimates of the value of some factors are going to be rough at best, only allowing them to be decisive ties reduces the chance of a misestimation and ensuing misallocation.^7^ There are problems with this line of argument. First, if the tiebreaking factor is truly valuable, we risk misallocation by not including it in our primary considerations anyway. We could end up ranking patients unfairly due to not taking a morally relevant factor into account. Second, the pragmatic reasons for including a factor as a tiebreaker must still outweigh our theoretical reasons for rejecting tiebreakers and our reasons for preferring a lottery. As we will argue in the next section, if a factor is truly only important enough to play the role of tiebreaker, it will not overcome competing reasons for preferring a lottery.

Furthermore, pragmatically motivated or not, the reason outlined above for using age as a tiebreaker rather than as a primary consideration was that we were somewhat unsure about the moral importance of age. While it is still difficult to understand why age is important enough to make a normative difference but only as a tiebreaker there is a more serious problem with this line of reasoning: the underlying motivation for using age as a tiebreaker (rather than a primary consideration) was that we wanted to give age a minimal weight. However, the result seems to be quite the opposite: while age was supposed to make a minimal difference, it seems as if it makes a maximum difference in the specific situation of a tie.

This issue is particularly problematic in some cases. As mentioned above, when introduced in the priority setting system in a lexical manner, the importance of the tiebreaker is dependent on our theoretical commitments. In systems where large numbers of options are likely to be tied, such as the Australian guidelines, the tiebreaker has a lot of practical significance. Accordingly, the point about tiebreakers being minimally valuable while making a large difference seems particularly counterintuitive in systems where ties would be common. Again, why should not a feature this practically significant be included among our primary considerations? To include the tiebreaking factor among the primary considerations would avoid the problem of tiebreakers being both minimally valuable (compared with the primary considerations) while still making a maximal difference in ties.

## Why not a lottery?

An alternative to the lexical tiebreaker is a lottery. Therefore, one may ask if the normative force of the (lexical) tiebreaker is stronger than the normative reasons for preferring a lottery. Prima facie, the rationale for preferring a lottery seems strongest precisely when there is no morally relevant difference between options; that is, when there is a tie. If we use tiebreakers when there are no morally relevant differences between outcomes, it seems unlikely that the value of the tiebreaker will outweigh reasons for preferring a lottery. We call this problem the second tiebreaker problem:

*Second tiebreaker problem*: if a tiebreaker is only significant when there are no morally relevant differences among options in terms of primary value, can it plausibly be significant enough to overcome reasons for preferring a lottery?

The second tiebreaker problem has a theoretically simple solution: deny that there is any normative reason for preferring a lottery. The minimal significance of the tiebreaker will then stand unopposed. While this is a theoretical option, it seems deeply counterintuitive. As we outlined above, lotteries have been argued for based on impartiality, fairness, respect for the separateness of persons and the moral value of chances.

To claim that there is no reason whatsoever for preferring a lottery amounts to saying that these normative reasons would amount to null even if unopposed. Taking this position seems connected to the following challenge: imagine that we have an exact tie among the primary considerations, and that the tiebreakers are also tied. For example, in the Australian guidelines there may be a tie between A and B in the sense that they both belong to the ‘high priority group’. However, it may well be the case that A and B have both been exposed to previous disadvantage, and they may both be medical professionals. Having rejected normative reasons for preferring a lottery, on what grounds should we make the choice between A and B? The only possibility now seems to be to add additional tiebreakers. As the number of additional tiebreakers that are being introduced increases the value ascribed to these secondary, tertiary, quaternary, etc values diminish. There is plausibly a limit to how small a value can be and still be considered as just that. Lacking any normative reasons for preferring a lottery, any non-defeated option (ie, an option that is not defeated due to being racist, sexist, ableist or similar) will possibly be on equal footing with the option of a lottery. This seems unacceptable.

While we have argued that lotteries are the superior option for breaking ties, they have problems of their own. It is beyond the scope of this paper to address the substantial theoretical literature on lotteries,^8^ but let us briefly sketch a practical concern. As Stone^10^ points out, people do not like lotteries. There is something disquieting about settling something as important as who gets a lifesaving resource by the luck of the draw. Requiring healthcare professionals to literally flip a coin in triage situations may cause moral stress, and they as well as the public might object.^9^ This is a real issue for the use of lotteries in priority setting. If lotteries are to be implemented, we should carefully consider how to do so in ways that are both minimally morally straining for healthcare workers and acceptable to the public. Since we have strong reasons for preferring lotteries as tiebreakers, this is an important future line of research.

## Conclusion

Our argument supports two conclusions about tiebreakers. First off, including tiebreaking factors as lexically lower ranked values in our priority setting system lacks a sound rationale, and it has the problematic result that a professed minimally valuable factor makes a massive difference in certain situations. This seems particularly problematic if ties are common in our priority setting system. Second, it seems unlikely that the tiebreaking factors, which we profess to be minimally valuable, can overcome competing reasons for using a lottery. Factors that we consider valuable should, therefore, all be included among the primary considerations, while ties should be settled by lotteries.

## Data Availability

No data are available.

## Footnotes

See McPherson,^11^ for an overview of this field.

While we take lotteries to be the most reasonable way to secure impartiality in ties, there may be alternatives. However, most of these alternatives comes with a price: the risk of introducing bias. For example, first come, first served may favour people with resourceful families. See Wasserman^12^ for a discussion of alternatives to lotteries in the standard sense.

See, for example, Kamm^13^ on the tension between utilitarian concerns and fairness and respect for the separateness of persons and Peterson^14^ for the independent moral value of chances.

The infinitesimal interpretation will have the same consequences as the lexical interpretation except if we are discussing infinite numbers, where the infinitesimal value of the tiebreaker could become relevant even with differences in primary considerations.

We would like to thank an anonymous reviewer for bringing this to our attention.

This is not to say that all morally relevant factors are pragmatically simple to account for. Measuring, for instance, desert may be highly difficult, and we may therefore risk misallocating resources. But, as we argue next, using such a pragmatically difficult factor as a tiebreaker also risks misallocation.

We would like to thank an anonymous reviewer for suggesting this argument.

But see, for instance, Broome,^7^ Wasserman^13^ and Stone^10^ for discussion on the moral properties of lotteries.

We would like to thank an anonymous reviewer for suggesting that we address this issue.
